# Association between intradialytic hypotension and physical function in patients undergoing maintenance hemodialysis: a multicenter cross-sectional study

**DOI:** 10.3389/fmed.2025.1655597

**Published:** 2025-10-29

**Authors:** Chun Qi Hou, Xi Ju Luo, Qin Juan Xu, Min Zhi, Min Liu, Si Yan Deng, Su Su Wang, Hua Gang Hu

**Affiliations:** ^1^Hemodialysis Center, The First Affiliated Hospital of Soochow University, Suzhou, Jiangsu, China; ^2^Department of Cardiovascular Medicine, Tongren People’s Hospital, Tongren, Guizhou, China; ^3^School of Nursing, Suzhou Medical College of Soochow University, Suzhou, Jiangsu, China; ^4^Department of Nephrology, Suzhou BenQ Medical Center, Suzhou, Jiangsu, China

**Keywords:** blood pressure, intradialytic hypotension, maintenance hemodialysis, physical function, nursing

## Abstract

**Background and objectives:**

The progressive decline in physical function is associated with a reduced quality of life and an increased risk of adverse clinical outcomes among patients undergoing maintenance hemodialysis (MHD). Intradialytic hypotension (IDH) has been identified as an independent risk factor for higher mortality and hospitalization rates in MHD patients. Nevertheless, the relationship between IDH and physical function in this population remains poorly understood. This study aimed to investigate the relationship between IDH and objectively assessed physical function in MHD patients.

**Methods:**

This cross-sectional study was carried out in five blood purification centers in Suzhou, China, from March to July 2023. Physical function was assessed using a battery of test, including the six-minute walk test (6MWT), the 10-repetition sit-to-stand-to-sit test (STS10), the 30-s sit-to-stand-to-sit test (STS30), handgrip strength (HGS), and the timed up-and-go test (TUG). Intradialytic blood pressure data were retrospectively extracted from the electronic medical record system prior to the physical function assessments, encompassing 36 consecutive hemodialysis sessions for each patient. Multiple linear and Poisson regression analyses were performed to explore the association between the frequency of IDH and physical function. This study adhered to the Strengthening the Reporting of Observational Studies in Epidemiology checklist for reporting observational research.

**Results:**

The study included 193 participants, of whom 67.9% were male, with a mean age of 53.3 years (SD = 12.6). The overall incidence of IDH was 13.7% across all hemodialysis sessions analyzed. After adjustment for potential confounders, multivariable regression analyses revealed that the higher frequency of IDH was significantly associated with poorer performance of the 6MWT (unstandardized coefficients [B] and 95% confidence interval [CI]: −2.715, −4.111 to −1.319 meters), the STS30 (−0.005, −0.009 to 0.000 repetitions), and HGS (−0.127, −0.236 to −0.018 kilograms). Conversely, more frequent IDH episodes were associated with the longer completion time in the STS10 (0.112, 0.004 to 0.220 s) and the TUG (0.033, 0.003 to 0.063 s).

**Conclusion:**

A significant inverse relationship was found between the higher IDH frequency and worse physical function in MHD patients, manifested by impaired walking ability, reduced upper and lower limb muscle strength, and diminished mobile balance ability. These findings suggest that optimized blood pressure management during hemodialysis to minimize IDH occurrence could potentially preserve physical function in this population.

## 1 Introduction

The incidence of end-stage renal disease (ESRD) has risen considerably in recent decades (1). Individuals with ESRD need renal replacement therapy, which can be delivered via dialysis or kidney transplantation, to sustain kidney function and ensure life-saving treatment (2). Maintenance hemodialysis (MHD) is the main form of renal replacement therapy (3). In China, data from the Chinese National Renal Data System (CNRDS) show that the number of MHD patients rose from 248,000 in 2012 to 844,000 in 2022 (4), and had further risen to 916,700 by 2024.

Physical function refers to a person’s ability to perform daily activities and tasks that involve movement and coordination, such as walking, muscle strength, balance, and cardiopulmonary capacity (5). Good physical function allows individuals to perform tasks like walking, climbing stairs, and maintaining overall independence in their daily lives. This is particularly important for MHD patients to maintain their daily activities and health-related quality of life (6). However, the physical functions of MHD patients often deteriorate significantly with extended dialysis treatment (7). Several factors contribute to this decline, including dialysis treatment, physical inactivity, cardiovascular abnormalities, comorbidities, anemia, and sarcopenia (8–10). Decreased physical function adversely affects overall health and quality of life, leading to a higher risk of hospitalization and mortality (5, 7). Therefore, it is crucial to identify modifiable risk factors that lead to the decline in physical function among MHD patients.

Intradialytic hypotension (IDH) is a serious complication for those undergoing MHD, defined as a decrease in blood pressure during dialysis or a nadir intradialytic blood pressure that falls below a certain level (11). The incidence of IDH varies widely, ranging from 7.5 to 69%, depending on the definitions used (12). IDH could leads to inadequate dialysis, and may impair the perfusion of important organs, such as the myocardium, brain, kidney, and bowel (13). A study involving 39,497 MHD patients found that IDH is associated with increased all-cause mortality (hazard ratio [HR] 1.07, 95% confidence interval [CI] 1.01–1.14), hospitalization (HR 1.13, 95%CI 1.08–1.18), and cardiovascular mortality (HR 1.10, 95%CI 1.03–1.17) (14).

Additionally, IDH is closely related to post-dialysis fatigue (15) and long dialysis recovery time (16). Post-dialysis fatigue is a frequent and one of the most debilitating symptoms affecting MHD patients and impairing their daily living and quality of life (17). IDH can impede effective dialysis and fluid removal, raising the risk of ischemia in vital organs such as the heart, brain, and gastrointestinal tract. Hence, the stress response caused by IDH may contribute to the development of post-dialysis fatigue (15). Gil et al. (18) enrolled 60 MHD patients to explore the relationship between the frequency of IDH and the degree of fatigue and recovery time from fatigue after dialysis. The findings revealed that a lower frequency of IDH is associated with decreased levels of post-dialysis fatigue (*r* = −0.39, *p* = 0.002) and quicker recovery time from post-dialysis fatigue (*r* = −0.36, *p* < 0.001) (18). Thus, exhaustion due to IDH likely discourages patients from engaging in physical activities, leading to a gradual decline in physical function over time. Moreover, IDH may impair lower extremity blood flow and aggravate limb ischemia. A retrospective observational study of 147 MHD patients indicated that the risk of critical limb ischemia in those with IDH was nearly three times higher than in those without IDH (HR = 3.13, *p* = 0.04) (19). Therefore, frequent IDH episodes may lead to insufficient blood supply to the limbs and reduced muscle strength.

The occurrence of IDH will increase the risk of post-dialysis fatigue, extend recovery time post-treatment, and reduces blood perfusion in the limbs, potentially resulting in a decrease in the physical function of MHD patients. Given the important of physical function for individuals, it is essential to understand the relationship between IDH and physical function in MHD patients. However, this relationship between IDH and physical function in MHD patients has not been fully elucidated. The aim of this study was to investigate the characteristics of IDH and to objectively assess the physical function of MHD patients, while also examining the associations between them. It is imperative to establish a stronger theoretical basis for future research on interventions aimed at preventing IDH in MHD patients and enhancing their physical function.

## 2 Materials and methods

### 2.1 Study design and ethics

This multicenter cross-sectional study was conducted in five blood purification centers in Suzhou, China, between March and July 2023. The study adhered to the Strengthening the Reporting of Observational Studies in Epidemiology (STROBE) statement (20). The study was conducted according to the guidelines of the Declaration of Helsinki, and approved by the Ethics Committee of Soochow University (SUDA20230115H01). All participants provided written informed consent prior to study enrollment.

### 2.2 Participants

Eligibility criteria for MHD patients included: (1) receiving MHD treatment for at least 3 months, (2) aged 18 years or older, (3) hemodialysis frequency of three times per week, (4) absence of surgical procedures within the preceding 3 months, and (5) volunteered to participate in this study. Exclusion criteria comprised: (1) diagnosed cognitive impairment or mental disorders, (2) impaired ambulation requiring assistive devices, (3) acute medical condition (e.g., active systemic infections and cardiovascular events) within the previous month, and (4) fever and infection events during the study.

### 2.3 Sample size calculation

The sample size was calculated using the formula: hemodialysis sessions = [Z_(1-*α*/2)_/d]^2^p(1−p) (21), where p represents the expected proportion of active IDH. Based on a previously published study, we assumed the expected proportion to be 14.4% (22), and we set the precision (d) at 0.01. With α = 0.05, Z_(1-α/2)_ = 1.96, resulting in a total of 4,735 hemodialysis sessions. To account for 20% incomplete data, a total of 5,919 hemodialysis sessions were necessary. Since we planned to include 36 sessions for each participant, we required 165 MHD patients.

### 2.4 Data collection and measurements

In each dialysis unit, a trained investigator conducted data collection. The investigator recruited MHD patients through interviews. After obtaining written consent, information was collected using the electronic medical record system (including blood pressure and disease-related data), and through in-person interviews with the participants (sociodemographic data). Physical function was assessed in all patients the day after a dialysis session.

#### 2.4.1 Sociodemographic data

The sociodemographic data collected included sex, age, body mass index (BMI), current employment status, and education level. Clinical characteristics, including dialysis vintage, type of blood purification, dialysis frequency, vascular access, use of antihypertensive agents pre-dialysis, etiology of ESRD, comorbidities (e.g., cardiovascular and cerebrovascular diseases, hypertension, and diabetes), and laboratory data (e.g., hemoglobin, albumin, calcium, phosphorus, creatinine levels, and dialysis adequacy measured by Kt/V), were extracted from the patients’ medical records. Additionally, exercise habits were recorded.

#### 2.4.2 Intradialytic blood pressure

Intradialytic blood pressure was extracted from the electronic medical record system for each participant 3 months before their physical function test, covering 36 consecutive dialysis sessions. The collection of blood pressure was accomplished by clinical staff in their daily work using an upper arm cuff sphygmomanometer (Model IHEM-780®, Omron Corporation) during hemodialysis. Blood pressure was measured every 60 min during each dialysis session. The nadir systolic blood pressure (SBP) during each dialysis session was utilized for data analysis.

#### 2.4.3 Intradialytic hypotension

IDH was defined as an intradialytic nadir SBP of less than 100 mmHg (11, 23), which is one of the most strongly related to the mortality of hemodialysis patients (24). The total number of sessions with IDH during the 36 dialysis sessions was recorded. Patients were categorized into the IDH group if they experienced IDH in 10% or more of their dialysis sessions (i.e., four sessions) (25). All other patients were classified into the non-intradialytic hypotension (no-IDH) group.

#### 2.4.4 Physical function: six-minute walk test

The six-minute walk test (6MWT) evaluates walking ability, balance, and lower limb muscle strength (26). The test was performed following the guidelines established by the American Thoracic Society (27). Participants were instructed to walk as fast as possible for 6 min on a 30-meter flat track, which was marked with bright strips every three meters. The distance covered was recorded in meters. The researcher used standardized instructions from the protocol to instruct and encourage participants during the walk. The 6MWT is a reliable and valid measure of walking capacity, cardiopulmonary function, and aerobic capacity, with a test–retest reliability (ICC) between 0.93 and 0.94 in MHD patients (28, 29). It has also been validated against peak oxygen uptake, showing moderate correlation coefficients (*r* = 0.62) in MHD patients (30).

#### 2.4.5 Physical function: 10-repetition sit-to-stand-to-sit test and 30-s sit-to-stand-to-sit test

The sit-to-stand-to-sit test (STS) was used to assess the lower limb muscle strength (10-repetition sit-to-stand-to-sit test, STS10) and endurance (30-s sit-to-stand-to-sit test, STS30) of the participants (31, 32). Participants were asked to stand up from a seated position and then sit back down, with their arms crossed over their chests, on a standard 44-centimeter straight-back chair without armrests. The time (in seconds) taken to complete 10 repetitions and the number of repetitions completed in 30 s were recorded. The STS10 was performed first, followed by a 10-min break before the STS30. Participants were encouraged to perform the tasks as quickly as possible, starting and finishing in a seated position. The STS30 is a reliable and valid measure for evaluating lower limb muscle strength, endurance, and function in MHD patients (33), with an ICC of 0.93 (29, 33).

#### 2.4.6 Physical function: handgrip strength

Handgrip strength (HGS) serves as a significant measure of upper limb muscle strength and nutritional status (34). It was measured in non-fistula hands by using a handgrip dynamometer (CAMRY-EH101, Yiwu Aolai Sports Goods Co., Ltd., China). Participants were first familiarized with the device and instructed to grip it with maximum strength while standing and keeping their elbows straight. The results were recorded in kilograms. At least two attempts were performed, the rest period between trials was at least 30 s, and the highest value was used for analysis. HGS is recognized as an independent predictor of all-cause mortality in MHD patients (35).

#### 2.4.7 Physical function: timed up-and-go test

The Timed Up-and-Go (TUG) test was used to assess the mobile balance ability of the participants (7). Each participant was seated alone in a standard chair that was 44 centimeters high, with their back against the chair, arms at their sides, and feet flat on the floor. They were instructed to stand up, walk three meters forward as quickly as possible, turn around an obstacle, return to the chair, and sit down again. The time taken to complete this task was recorded in seconds. Two sets of testing were performed, with a 60-s rest interval in between, and the best result was selected for analysis. The TUG is considered a reliable and valid assessment of balance in MHD patients, with an ICC of 0.91 (36).

### 2.5 Statistical analysis

First, the Kolmogorov–Smirnov test along with the quantile-quantile (Q-Q) plot was used to determine if each continuous variable followed a normal distribution. Second, continuous data that met the normality distribution were presented as means with standard deviations (SD), while skewed data were presented as medians with interquartile ranges (IQR). Categorical data were shown as numbers (percentages). Third, an independent samples *t*-test was used to compare physical function between the IDH and no-IDH groups. Further, *Cohen’s d* effect size was calculated by finding the mean difference between the groups and dividing it by the pooled SD, with classifications as small effect size (*d* = 0.2), moderate effect size (*d* = 0.5), and large effect size (*d* = 0.8) (37). Fourth, the correlation between quantitative data was examined using Pearson’s correlation for continuous variables that met normal distribution, and Spearman’s correlation for those that were not. Fifth, the relationship between the frequency of IDH and physical function in MHD patients was examined using three linear regression models or Poisson regression models. The STS30 is a count variable and follows a Poisson distribution; therefore, it was analyzed with a Poisson regression model. All other indices are continuous numerical variables and were analyzed using linear regression models. Physical function served as the dependent variable, while the independent variable in the crude model was the frequency of IDH within 36 dialysis sessions. To control for potential sociodemographic and disease-related confounders, multivariate regression models (model 1 and 2) were conducted. Model 1 included sociodemographic factors (i.e., sex, age, BMI, dialysis vintage, employment status, and education level). Model 2 added disease-related factors (i.e., comorbidities, type of blood purification, hemoglobin, albumin, phosphorus, and creatinine) and if regular exercise based on model 1. In the multiple linear regression model, we included the ‘frequency of IDH episodes’ as a continuous independent variable and assumed a linear relationship with the dependent variable. A locally weighted scatterplot smoothing (LOWESS) plot indicated an approximately linear relationship between the variables, and residual analysis of the model detected no apparent nonlinear patterns, thus supporting the use of a linear term. Model covariates were selected based on published studies (8–10) and univariate analyses. More importantly, the variance inflation factor (VIF) is less than 10. To validate the assumptions of the linear regression model, we performed a residual analysis. The residuals were plotted against the predicted values to assess linearity and homoscedasticity. The normality of residuals was examined using a normal Q-Q plot. No evident patterns were observed in the residual vs. fitted plot, suggesting that the assumptions of linearity and homoscedasticity were met. The points in the Q-Q plot approximately followed the straight line, indicating no substantial deviation from normality. The effect size was reported as unstandardized coefficients (B) with 95% CI, and the *R^2^* value was used to evaluate the goodness-of-fit for the linear regression model. The pseudo-*R^2^* value was used to evaluate the goodness-of-fit for the Poisson regression model. Adopting the Multiple Imputation by Chained Equations (MICE) method to handle missing data, generating 10 imputed datasets (m = 10). The imputation model is based on multivariate linear regression and predictive mean matching (PMM). In the analysis phase, Cox regression analysis was conducted on each imputed dataset, and the results were pooled using Rubin’s rules. The specific imputation variable is mainly albumin. Analyses were performed using SPSS software (version 26.0). A two-sided *p* value of less than 0.05 was considered statistically significant.

## 3 Results

### 3.1 Study patients

**Figure 1:**
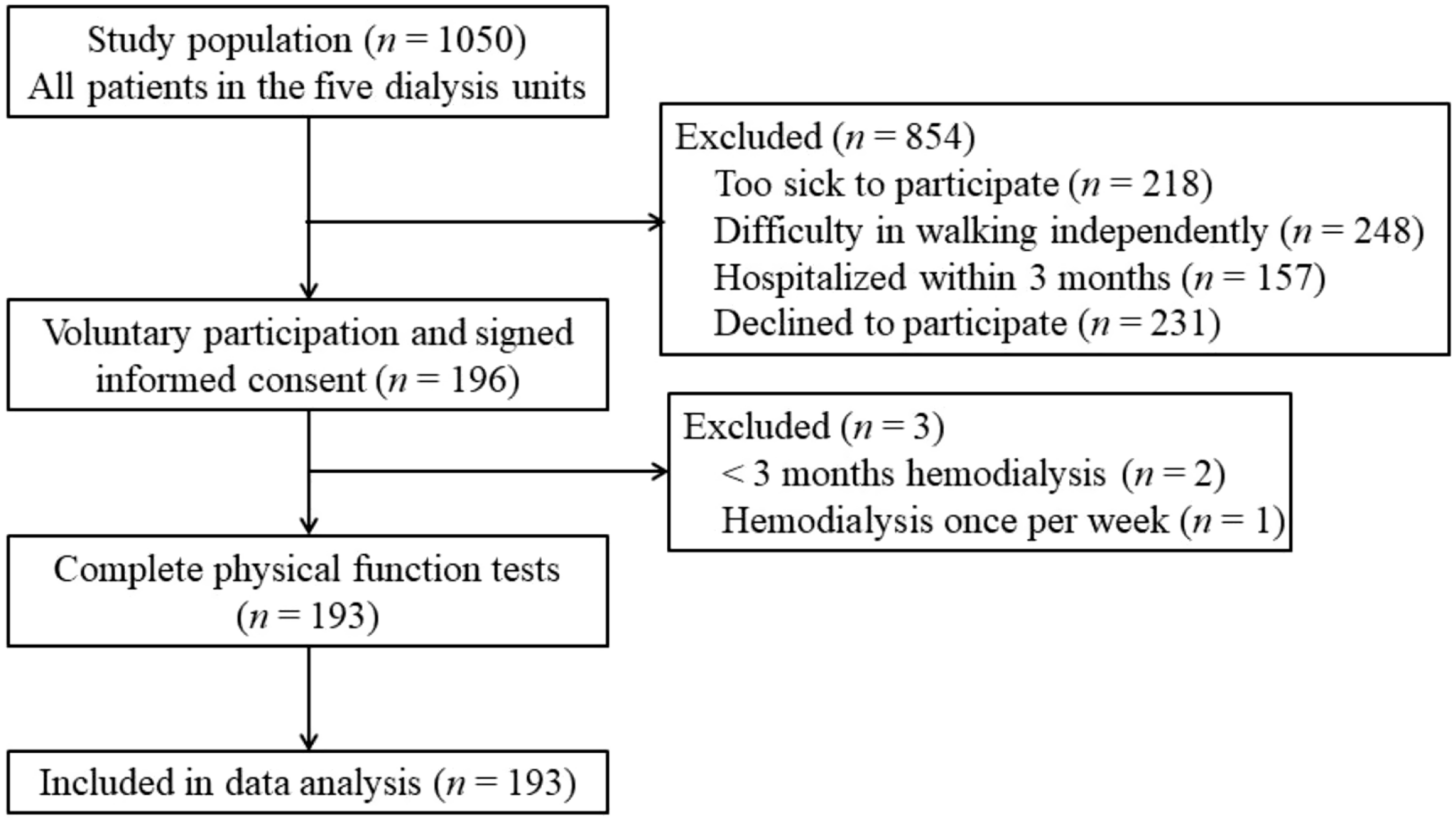
Flow chart for the cross-sectional study.

A total of 193 participants (67.9% male) and 6,948 dialysis sessions were analyzed (Figure 1). The average age of the participants was 53.3 years (SD = 12.6), and the mean dialysis vintage was 64.8 months (SD = 52.9). The incidence of IDH was 13.7% across all studied dialysis sessions, with 56 individuals (29.0%) classified in the IDH group. Comparisons between the groups showed significant differences in sex, education level, dialysis vintage, use of antihypertensive agents pre-dialysis, hemoglobin, and Kt/V between the IDH and no-IDH groups (*p* < 0.05). The sociodemographic and disease-related characteristics are summarized in Table 1.

**Table 1:** Characteristics of maintenance hemodialysis patients (*n* = 193).

Characteristics	Total (*n* = 193)	no-IDH group (*n* = 137)	IDH group (*n* = 56)	*t*/*χ^2^*	*p* value
Sex				9.367^a^	0.002
Male	131 (67.9)	102 (74.5)	29 (51.8)		
Female	62 (32.1)	35 (25.5)	27 (48.2)		
Age (years), mean (SD)	53.3 (12.6)	52.6 (12.8)	54.9 (12.0)	−1.135^b^	0.258
BMI (kg/m^2^), mean (SD)	21.8 (3.3)	21.7 (3.2)	22.1 (3.7)	−0.745^b^	0.457
Current employment				3.436^a^	0.064
No	133 (68.9)	89 (65.0)	44 (78.6)		
Yes	60 (31.1)	48 (35.0)	12 (21.4)		
Education level				4.140^a^	0.042
Below high school	102 (52.8)	66 (48.2)	36 (64.3)		
High school or above	91 (47.2)	71 (51.8)	20 (35.7)		
If regular exercise				0.069^a^	0.793
No	56 (29.0)	39 (28.5)	17 (30.4)		
Yes	137 (71.0)	98 (71.5)	39 (69.6)		
Type of blood purification				0.037^a^	0.848
HD	22 (11.4)	16 (11.7)	6 (10.7)		
HD + HDF or HD + HP	171 (88.6)	121 (88.3)	50 (89.3)		
Dialysis vintage (months), mean (SD)	64.8 (52.9)	54.9 (49.2)	88.9 (54.4)	−4.219^b^	< 0.001
Vascular access				0.044^a^	0.833
Arteriovenous fistula	182 (94.3)	130 (94.9)	52 (92.9)		
Central venous catheter	11 (5.7)	7 (5.1)	4 (7.1)		
Using antihypertensive agents pre-dialysis				28.400^a^	< 0.001
No	137 (71.0)	82 (59.9)	55 (98.2)		
Yes	56 (29.0)	55 (40.1)	1 (1.8)		
Etiology of ESRD				7.115^a^	0.310
Glomerular nephritis	100 (51.8)	64 (46.7)	36 (64.3)		
Hypertension	8 (4.1)	7 (5.1)	1 (1.8)		
Diabetic nephropathy	15 (7.8)	11 (8.0)	4 (7.1)		
Polycystic kidney	16 (8.3)	11 (8.0)	5 (8.9)		
Obstructive nephropathy	6 (3.1)	4 (2.9)	2 (3.6)		
IgA nephropathy	6 (3.1)	5 (3.6)	1 (1.8)		
Drug-induced nephritis or unknown	42 (21.8)	35 (25.5)	7 (12.5)		
If comorbidity				0.178^a^	0.673
No	68 (35.2)	47 (34.3)	21 (37.5)		
Yes	125 (64.8)	90 (65.7)	35 (62.5)		
Hemoglobin (g/L), mean (SD)	112.1 (16.3)	110.1 (16.2)	117.0 (15.6)	−2.708^b^	0.007
Albumin (g/L), mean (SD)	41.5 (3.5)	41.5 (3.6)	41.5 (3.3)	−0.052^b^	0.959
Calcium (mmol/L), mean (SD)	2.3 (0.2)	2.2 (0.2)	2.3 (0.3)	−1.214^b^	0.226
Phosphorus (mmol/L), mean (SD)	2.0 (0.5)	2.0 (0.5)	1.9 (0.6)	1.030^b^	0.304
Creatinine (μmol/L), mean (SD)	1030.5 (278.8)	1039.8 (292.4)	1007.6 (243.4)	0.729^b^	0.467
Kt/V, mean (SD)	1.4 (0.4)	1.3 (0.4)	1.5 (0.4)	−2.764^b^	0.006

Data are given as numbers (percentages) unless stated otherwise.

^a^Represents χ^2^ value, ^b^ represents t value.

BMI body mass index, HD hemodialysis, HDF hemodiafiltration, HP hemoperfusion, ESRD end-stage renal disease, IDH intradialytic hypotension, SD standard deviation.

### 3.2 Physical function on maintenance hemodialysis patients with and without intradialytic hypotension

Table 2 displays the physical function of MHD patients. The Kolmogorov–Smirnov test and Q-Q plot test showed that the study’s outcome measures followed a normal distribution. The independent samples *t*-test indicated significant differences in the 6MWT (*d* = 0.485) and HGS (*d* = 0.464) between the no-IDH and IDH groups (all *p* < 0.05), reflecting small to moderate effect sizes. In contrast, the differences in the STS10, STS30, and TUG between the two groups were insignificant (*p* > 0.05).

**Table 2:** The physical function in difference intradialytic hypotension group on maintenance hemodialysis patients by independent samples *t*-test.

Physical function	Total	no-IDH group	IDH group	*Cohen’s d*	*p* value
6MWT (meters)	465.2 (96.3)	478.4 (89.5)	432.7 (105.4)	0.485	0.003
STS10 (seconds)	21.8 (6.0)	21.4 (5.8)	22.8 (6.4)	0.233	0.192
STS30 (repetitions)	16.9 (4.7)	17.1 (4.7)	16.5 (4.5)	0.130	0.422
HGS (kilograms)	31.4 (9.8)	32.7 (9.6)	28.2 (9.7)	0.464	0.004
TUG (seconds)	7.6 (1.7)	7.5 (1.6)	7.8 (1.8)	0.190	0.279

Data are given as mean (standard deviation).

6MWT six-minute walk test, STS10 10-repetition sit-to-stand-to-sit test, STS30 30-s sit-to-stand-to-sit test, HGS handgrip strength, TUG timed up-and-go test, IDH intradialytic hypotension.

### 3.3 Physical function in maintenance hemodialysis patients with different characteristics

The comparison between groups showed significant differences in physical function among MHD patients related to sex, current employment status, education level, and exercise habits (*p* < 0.05) (Supplementary Table S1). Additionally, correlation analysis found that age, BMI, albumin, phosphorus, and creatinine levels were associated with the physical function in MHD patients (*p* < 0.05) (Supplementary Table S2).

### 3.4 Association between the frequency of intradialytic hypotension and physical function in maintenance hemodialysis patients

The results of the multivariate regression model are summarized in Table 3. After adjusting for possible confounding variables, the multivariate regression analyses revealed that a higher frequency of IDH was significantly associated with lower levels of the 6MWT (B and 95% CI: −2.715, −4.111 to −1.319 meters), the STS30 (B and 95% CI: −0.005, −0.009 to 0.000 repetitions), and HGS (B and 95% CI: −0.127, −0.236 to −0.018 kilograms). In contrast, a higher frequency of IDH was significantly positively associated with the STS10 (B and 95% CI: 0.112, 0.004 to 0.220 s) and the TUG (B and 95% CI: 0.033, 0.003 to 0.063 s).

**Table 3:** The relationship between the frequency of intradialytic hypotension and physical function on maintenance hemodialysis patients by multivariate regression analyses (multiple linear regression or Poisson regression).

Physical function	Crude model			Model 1			Model 2		
	*R^2^*/*Pseudo R^2^*	B (95% CI)	*p* value	*R^2^*/*Pseudo R^2^*	B (95% CI)	*p* value	*R^2^*/*Pseudo R^2^*	B (95% CI)	*p* value
6MWT^a^	0.095	−3.175 (−4.580, −1.769)	< 0.001	0.296	−2.590 (−3.996, −1.185)	< 0.001	0.374	−2.715 (−4.111, −1.319)	< 0.001
STS10^a^	0.023	0.101 (−0.006, 0.207)	0.063	0.263	0.089 (−0.018, 0.196)	0.103	0.321	0.112 (0.004, 0.220)	0.043
STS30^b^	0.003	−0.004 (−0.008, 0.000)	0.079	0.042	−0.004 (−0.008, 0.001)	0.120	0.070	−0.005 (−0.009, 0.000)	0.039
HGS^a^	0.062	−0.263 (−0.409, −0.117)	< 0.001	0.566	−0.114 (−0.226, −0.001)	0.047	0.638	−0.127 (−0.236, −0.018)	0.023
TUG^a^	0.052	0.041 (0.013, 0.069)	0.004	0.219	0.033 (0.003, 0.063)	0.029	0.279	0.033 (0.003, 0.063)	0.029

The crude model was adjusted for no covariates.

The model 1 was adjusted for sex, age, body mass index, dialysis vintage, work status, education level.

The model 2 was adjusted for sex, age, body mass index, dialysis vintage, work status, education level, if regular exercise, comorbidity, type of blood purification, hemoglobin, albumin, phosphorus, and creatinine.

^a^Multivariate linear regression model; ^b^ multivariate Poisson regression model.

6MWT six-minute walk test, STS10 10-repetition sit-to-stand-to-sit test, STS30 30-s sit-to-stand-to-sit test, HGS handgrip strength, TUG timed up-and-go test, CI confidence interval.

## 4 Discussion

This research shows a significant relationship between the occurrence of IDH and low levels of physical function, specifically in terms of walking ability, upper and lower extremity muscle strength, and mobility balance in MHD patients. Consequently, preventing IDH is essential for this population. Additionally, more focus should be placed on the lowest blood pressure observed during dialysis, as it could signal negative outcomes for MHD patients.

Despite the important clinical significance of IDH in MHD patients, there is still no consensus on its definition (11). The National Kidney Foundation’s Kidney Disease Outcomes Quality Initiative (KDOQI) guidelines recommendation characterize IDH as a drop in SBP ≥ 20 mmHg, or a mean arterial pressure ≥ 10 mmHg, along with related symptoms (38). On the other hand, the European Best Practice Guideline (EBPG) describes IDH as a situation associated with a clinical event that requires nursing intervention (39). In China, the Intradialytic Hypotension Prevention and Treatment Expert Working Group recommended the KDQOI and EBPG definition of IDH and suggested refusing to use the absolute value of intradialytic blood pressure as the diagnostic criterion for IDH (40). However, we may need to reconsider the definition of IDH for the following reasons. In China, pre-dialysis blood pressure is not a compulsory data in routine dialysis work currently. As a result, it may be impossible to assess the drop in blood pressure from pre-dialysis readings for patients whose pre-dialysis blood pressure was not documented. This gap could lead to missed chances to monitor patients who might be at risk due to low intradialytic blood pressure. A large cohort study including 11,801 hemodialysis patients from two dialysis organizations in Europe found that IDH, defined as an intradialytic nadir SBP below 100 mmHg, was strongly associated with one-year mortality (odds ratio [OR] = 1.23, 95% CI = 1.05 to 1.44) (24). In addition, Zhi et al. included 479 hemodialysis patients and found that IDH (intradialytic nadir SBP < 100 mmHg) was associated with physical functioning dimensions of health-related quality of life (OR = −0.415, 95% CI = −0.685 to −0.144) (23).

In this study, the prevalence of IDH was 13.7 and 29.0% in terms of dialysis sessions and hemodialysis patients, respectively. Zhi et al. analyzed a total of 17,892 dialysis sessions from 497 participants. According to this study’s IDH definition (the lowest intradialytic SBP < 100 mmHg), 1,986 dialysis sessions experienced IDH, representing 11.1% of dialysis sessions. Among the 497 patients, 124 (24.9%) of them were assigned to the IDH group (23). The results are basically consistent with those of this study. Hara et al. included 545 MHD patients and analyzed 3,261 dialysis sessions, finding that IDH (defined as an intradialytic nadir SBP < 100 mmHg) occurred in 14.4% of the sessions (22), which is consistent with this study’s results. The univariate analysis indicated that MHD patients who were female, had longer dialysis vintage, lower educational attainment, use of antihypertensive drugs before dialysis, higher hemoglobin levels, and had a high Kt/V were more likely to develop IDH. He et al. included 3,906 MHD patients to predict the risk of IDH, finding that female patients were more likely to reported IDH than male patients (OR = 1.280, *p* < 0.001) (41), aligning with this study’s findings. Furthermore, a cross-sectional study showed that the risk of IDH increased by 1% for each month of dialysis vintage (OR = 1.01, *p* = 0.048), and the hemoglobin level was positively correlated with the frequency of IDH occurrences (B = 0.22, *p* = 0.021) (42), which further corroborates the results of this study.

The 6MWT is one of the critical outcomes preferentially considered by MHD patients (43). A cross-sectional study found that MHD patients had significantly lower 6MWT distances compared to age-matched healthy controls (483.0 [SD = 56.9] vs. 575.3 [SD = 43.3] meters, *p* < 0.001) (44). The average 6MWT of this study was 465.2 (SD = 96.3) meters, aligning with results from other studies (44). Furthermore, the research indicated that the 6MWT for patients in the IDH group was about 46 meters shorter than that of the no-IDH group (*d* = 0.485). Considering that the minimal clinically important difference (MCID) for the 6MWT ranges from 14.0 to 30.5 meters in similar patient groups (28, 45), we estimate the effect size to be moderate to large. The difference is clinically meaningful. Multiple linear regression analysis showed that, among the 36 dialysis sessions, the 6MWT decreased by 2.715 meters for each additional instance of IDH. An increase in the number of IDH episodes was correlated with a decline in walking capacity among MHD patients.

The HGS and STS test were employed to assess the upper and lower extremity muscle strength (peripheral muscle strength) of MHD patients, respectively. The study reported average results for HGS, STS10 and STS30 as 31.4 (SD = 9.8) kilograms, 21.8 (SD = 6.0) seconds and 16.9 (SD = 4.7) repetitions, respectively. Previous research has shown similar physical function results regarding HGS and STS compared to this study (9, 46). One study involving 102 MHD patients reported average HGS and STS10 values of 30.5 kilograms and 26.0 s, respectively (9). Another study with 72 MHD patients indicated a mean STS30 of 15.9 (SD = 5.3) repetitions (46). Furthermore, the research indicated that the HGS for patients in the IDH group was about 4.5 kilograms lower than that of the no-IDH group (d = 0.464). Considering that the MCID for the HGS was 3.4 kilograms in similar patient groups (28), we estimate the effect size to be moderate to large. The difference is clinically meaningful. Considering that the MCID for the STS10 was 8.4 s (28) and the STS30 was 2.1 repetitions (33) in similar patient groups. However, the research indicated that the STS10 for patients in the IDH group was about 1.4 s longer than that of the no-IDH group, the STS30 for patients in the IDH group was about 0.6 repetitions fewer than that of the no-IDH group. Therefore, the difference between the two groups in STS was not considered clinically significant. The findings from multiple linear regression analysis revealed that during the 36 dialysis sessions, each additional occurrence of IDH was associated with a decrease in HGS of 0.127 kilograms, an increase in STS10 of 0.112 s, and a decrease in STS30 of 0.005 repetitions. A higher frequency of IDH episodes was associated with reduced muscle strength in both the upper and lower extremities among MHD patients.

The TUG test evaluates a person’s mobile balance ability, which are crucial for patients to maintain a stable posture and carry out daily tasks (47). In this study, the mean TUG was 7.6 (SD = 1.7) seconds. A cross-sectional study found that the average TUG for 65 MHD patients was 8.8 s (48), which closely aligns with the results of this study. The research indicated that the TUG for patients in the IDH group was about 0.3 s longer than that of the no-IDH group. Considering that the MCID for the TUG was 2.9 s in similar patient groups (49). Therefore, the difference between the two groups in TUG was not considered clinically significant. The multiple linear regression analysis showed that during the 36 dialysis sessions, the TUG increased by 0.033 s for each additional instance of IDH. A higher frequency of IDH episodes was associated with poorer mobility and balance in MHD patients.

Our study demonstrated that a high frequency of IDH was significantly correlated with lower levels of physical function, specifically in terms of walking ability, upper and lower extremity muscle strength, and mobile balance ability in MHD patients. Therefore, preventing the occurrence of IDH may positively impact on the physical function of MHD patients. A meta-analysis involving 347 MHD patients found that the use of blood volume biofeedback systems reduces the risk of IDH (OR = 0.63, *p* = 0.03) (50). Various studies have indicated that intradialytic exercise can decrease the incidence of IDH in MHD patients (13, 51). Expert consensus on the prevention and treatment of IDH have indicated that strategies such as withholding or reducing antihypertensive drugs on dialysis days, limiting intradialytic food intake, improving patients’ nutritional status, and controlling inter-dialytic weight gain can effectively prevent IDH (40). Consistent with this expert opinion, our study identified several factors associated with a higher likelihood of IDH, including female sex, longer dialysis vintage, lower educational attainment, pre-dialytic use of antihypertensive drugs, higher hemoglobin levels, and a high Kt/V. Consequently, healthcare providers in dialysis centers should implement proactive measures, such as promoting intradialytic exercise and optimizing nutritional support, to mitigate IDH risk and thereby improve physical function in these patients. However, the cross-sectional design cannot establish a causal relationship between IDH and physical function, and it is unable to rule out time-related confounding factors. Therefore, the reduction of physical function in MHD patients may also lead to frequent IDH.

Hattori et al. (52) conducted a study involving 192 MHD patients to investigate how intradialytic blood pressure affects physical activity after dialysis. The findings revealed that a lower intradialytic nadir diastolic blood pressure was significantly associated with decreased physical activity. Physical inactivity leads to muscle weakness, exercise intolerance, and a poorer health-related quality of life (8). Another study showed that IDH might lead to further deterioration in physical function among MHD patients; however, most participants in that study were older adults, averaging 71 years old. Thus, it is uncertain if these results are applicable to a younger and more active population (52). In contrast, the participants in our study had a mean age of 53 years, which may better reflect the general MHD population.

This study expanded the knowledge of IDH and its prognostic outcomes in MHD patients, highlighting the necessity to prevent its occurrence. Our results revealed an inverse relationship between the occurrence of IDH and the objectively measured physical function in MHD patients. We identified several potential mechanisms by which IDH could lead to reduced physical function in this population. First, IDH is closely related to post-dialysis fatigue (15) and long dialysis recovery time (16). Post-dialysis fatigue is a frequent and one of the most debilitating symptoms affecting MHD patients and impairing their daily living and quality of life (17). IDH can impede effective dialysis and fluid removal, raising the risk of ischemia in vital organs such as the heart, brain, and gastrointestinal tract. Hence, the stress response caused by IDH may contribute to the development of post-dialysis fatigue (15). Findings from a multicenter prospective crossover study indicated that fewer instances of IDH were associated with lower levels of post-dialysis fatigue and quicker recovery times (18). Thus, exhaustion due to IDH likely discourages patients from engaging in physical activities, leading to a gradual decline in physical function over time. Second, IDH can lead to reduced blood flow to the brain, which may cause cerebral ischemia and increase the risk of depression (53). Garcia et al. (9) found a significant association between physical function and depression in MHD patients, suggesting that IDH could negatively impact physical function. Third, IDH may impair lower extremity blood flow and aggravate limb ischemia. A retrospective cohort study identified IDH as an independent risk factor for critical limb ischemia in MHD patients (HR = 3.13, *p* = 0.04) (19). Therefore, frequent IDH episodes may lead to insufficient blood supply to the limbs and reduced muscle strength.

However, the cross-sectional nature of this study limits its ability to determine causation, and it is possible that reduced physical function, especially lower muscle strength, may lead to low blood pressure or a drop in blood pressure during dialysis. Studies have shown that exercising during dialysis can significantly reduce the occurrence of IDH (54). This effect may be due to the activation of receptors in muscles and joints, which sends nerve signals that improve heart contractions, thereby increasing cardiac output and helping to prevent IDH. As a result, patients with decreased muscle strength might be more susceptible to developing IDH. Therefore, in clinical settings, MHD patients should be encouraged to participate in regular exercises during or outside of dialysis sessions, such as cycling in bed, walking, or jogging.

This research has several notable strengths. Firstly, it is the first study to investigate the relationship between the frequency of IDH and physical function in MHD patients. The findings can provide valuable insights for medical staff in blood purification centers. Secondly, objective assessments of physical performance were used to assess the physical function of MHD patients, resulting in findings that are more reliable than those derived from self-reported data. Lastly, multiple regression models were used to analyze the association between IDH and physical function, effectively minimizing potential confounders. However, there are also several limitations to this study. Firstly, all patients were from Suzhou, which may introduce regional bias, and future multi-regional studies are recommended. Secondly, the cross-sectional study design precluded the causal inferences between IDH and physical function. Thirdly, the lack of pre-dialysis blood pressure data meant that the study could not utilize the IDH definition provided in the guidelines. Nonetheless, this limitation enabled us to gain insights beyond just blood pressure fluctuations and related symptoms. Furthermore, the intradialytic nadir blood pressure warrants careful consideration.

## 5 Conclusion

Walking ability, upper and lower limb muscle strength, and mobile balance ability were significantly negatively associated with the frequency of IDH in MHD patients. Preventing the occurrence of IDH is essential for this population. Further studies are required to investigate the causal relationship and dose–response effects between IDH and physical function. A prospective multicenter trial with large cohorts is necessary to confirm the causal relationship between IDH and physical function.

## Data Availability

The datasets generated and/or analyzed during the current study are available from the corresponding author on reasonable request.
