# Supplementary material for: Association between intradialytic hypotension and physical function in patients undergoing maintenance hemodialysis: a multicenter cross-sectional study
Source: Front Med (Lausanne). 2025 Oct 29;12:1655597. doi: 10.3389/fmed.2025.1655597 (PMC12605339; doi:10.3389/fmed.2025.1655597)
Supplement: Supplementary file 1 [file Table_1.docx]

Table S1 The physical function on maintenance hemodialysis patients with different characteristics by independent samples *t*-test or analysis of variance

| Characteristics | 6MWT | STS10 | STS30 | HGS | TUG |
| --- | --- | --- | --- | --- | --- |
| Sex |  |  |  |  |  |
| Male | 479.5 (90.9) | 21.9 (6.5) | 17.0 (4.9) | 35.8 (8.6) | 7.4 (1.6) |
| Female | 435.6 (101.1) | 21.7 (4.8) | 16.7 (4.1) | 22.3 (4.6) | 7.8 (1.8) |
| *Cohen’s d* | 0.466 | 0.039 | 0.067 | 1.787 | 0.243 |
| *P* value | 0.003 | 0.825 | 0.667 | < 0.001 | 0.158 |
| Current employment |  |  |  |  |  |
| No | 447.2 (100.0) | 23.0 (5.9) | 16.0 (4.2) | 29.3 (9.2) | 7.9 (1.7) |
| Yes | 504.6 (74.5) | 19.5 (5.4) | 18.8 (5.1) | 36.1 (9.7) | 6.9 (1.4) |
| *Cohen’s d* | 0.619 | 0.620 | 0.624 | 0.731 | 0.584 |
| *P* value | < 0.001 | < 0.001 | < 0.001 | < 0.001 | < 0.001 |
| Education level |  |  |  |  |  |
| Below high school | 444.3 (100.9) | 22.7 (5.5) | 15.9 (4.0) | 29.1 (9.0) | 8.0 (1.8) |
| High school or above | 488.2 (85.8) | 20.7 (6.4) | 18.1 (5.1) | 34.0 (10.2) | 7.0 (1.3) |
| *Cohen’s d* | 0.466 | 0.331 | 0.471 | 0.507 | 0.567 |
| *P* value | 0.002 | 0.047 | 0.002 | < 0.001 | < 0.001 |
| Type of blood purification |  |  |  |  |  |
| HD | 432.3 (107.5) | 23.8 (7.1) | 15.6 (4.1) | 27.0 (9.7) | 8.0 (2.0) |
| HD + HDF or HD + HP | 469.5 (94.3) | 21.6 (5.8) | 17.1 (4.7) | 32.0 (9.7) | 7.5 (1.6) |
| *Cohen’s d* | 0.388 | 0.383 | 0.318 | 0.512 | 0.314 |
| *P* value | 0.088 | 0.120 | 0.171 | 0.025 | 0.192 |
| Vascular access |  |  |  |  |  |
| Arteriovenous fistula | 466.6 (97.3) | 21.7 (6.1) | 17.0 (4.7) | 31.8 (9.9) | 7.5 (1.7) |
| Central venous catheter | 442.2 (79.2) | 23.5 (3.7) | 16.2 (3.8) | 25.4 (6.4) | 8.0 (1.6) |
| *Cohen’s d* | 0.253 | 0.289 | 0.171 | 0.657 | 0.291 |
| *P* value | 0.416 | 0.379 | 0.584 | 0.036 | 0.374 |
| Using antihypertensive agents pre-dialysis |  |  |  |  |  |
| No | 464.9 (99.9) | 21.8 (5.7) | 17.1 (4.7) | 30.4 (9.5) | 7.6 (1.6) |
| Yes | 466.1 (87.7) | 21.9 (6.6) | 16.4 (4.6) | 33.8 (10.3) | 7.4 (1.8) |
| *Cohen’s d* | 0.013 | 0.007 | 0.147 | 0.350 | 0.127 |
| *P* value | 0.937 | 0.969 | 0.364 | 0.028 | 0.465 |
| Etiology of ESRD |  |  |  |  |  |
| Glomerular nephritis | 476.4 (94.7) | 21.4 (6.2) | 17.4 (4.4) | 32.9 (9.6) | 7.4 (1.5) |
| Hypertension | 470.3 (106.0) | 22.0 (7.8) | 16.3 (4.0) | 27.7 (8.9) | 7.9 (3.3) |
| Diabetic nephropathy | 411.8 (94.6) | 23.9 (4.2) | 13.6 (3.5) | 28.3 (7.6) | 7.7 (1.5) |
| Polycystic kidney | 477.3 (43.0) | 22.7 (3.6) | 15.3 (3.3) | 30.1 (9.2) | 7.3 (1.1) |
| Obstructive nephropathy | 459.5 (68.5) | 20.6 (6.8) | 18.5 (7.7) | 31.1 (7.8) | 7.5 (1.8) |
| IgA nephropathy | 429.7 (96.9) | 21.8 (5.2) | 16.2 (3.4) | 21.5 (1.7) | 8.5 (2.3) |
| Drug-induced nephritis or unknown | 468.6 (97.2) | 21.8 (6.5) | 17.9 (6.3) | 32.4 (11.8) | 7.3 (1.3) |
| *F* | 1.065 | 0.380 | 1.706 | 1.847 | 0.065 |
| *P* value | 0.386 | 0.891 | 0.124 | 0.094 | 0.726 |
| If comorbidity |  |  |  |  |  |
| No | 466.2 (105.8) | 21.3 (6.4) | 17.5 (5.4) | 30.6 (9.7) | 7.7 (2.0) |
| Yes | 464.7 (91.1) | 22.1 (5.8) | 16.6 (4.2) | 31.9 (9.9) | 7.5 (1.5) |
| *Cohen’s d* | 0.016 | 0.139 | 0.200 | 0.127 | 0.116 |
| *P* value | 0.918 | 0.418 | 0.194 | 0.400 | 0.491 |
| If regular exercise |  |  |  |  |  |
| No | 443.3 (87.4) | 22.2 (4.9) | 16.2 (4.0) | 31.5 (9.9) | 7.8 (1.7) |
| Yes | 474.1 (98.6) | 21.7 (6.3) | 17.3 (4.9) | 31.4 (9.9) | 7.5 (1.7) |
| *Cohen’s d* | 0.322 | 0.077 | 0.236 | 0.018 | 0.206 |
| *P* value | 0.046 | 0.683 | 0.140 | 0.909 | 0.281 |

Notes: Data are given as mean (standard deviation)

*Abbreviations: 6MWT* six-minute walk test, *STS10* 10-repetition sit-to-stand-to-sit test, *STS30* 30-second sit-to-stand-to-sit test, *HGS* handgrip strength, *TUG* timed up-and-go test, *HD* hemodialysis, *HDF* hemodiafiltration, *HP* hemoperfusion, *ESRD* end-stage renal disease

Table S2 Correlation between characteristics and physical function on maintenance hemodialysis patients by Pearson’s correlation or Spearman’s correlation analysis

| Characteristics | 6MWT | STS10 | STS30 | HGS | TUG |
| --- | --- | --- | --- | --- | --- |
| Age |  |  |  |  |  |
| Correlation coefficient | -0.382 ^a^ | 0.477 ^a^ | -0.416 ^b^ | -0.354 ^a^ | 0.396 ^a^ |
| *P* value | < 0.001 | < 0.001 | < 0.001 | < 0.001 | < 0.001 |
| BMI |  |  |  |  |  |
| Correlation coefficient | -0.223 ^a^ | 0.212 ^a^ | -0.133 ^b^ | 0.231 ^a^ | 0.087 ^a^ |
| *P* value | 0.002 | 0.009 | 0.069 | 0.001 | 0.279 |
| Dialysis vintage |  |  |  |  |  |
| Correlation coefficient | -0.017 ^a^ | -0.017 ^a^ | 0.078 ^b^ | -0.019 ^a^ | 0.045 ^a^ |
| *P* value | 0.821 | 0.834 | 0.288 | 0.797 | 0.582 |
| Hemoglobin |  |  |  |  |  |
| Correlation coefficient | 0.049 ^a^ | -0.051 ^a^ | 0.113 ^b^ | 0.100 ^a^ | 0.007 ^a^ |
| *P* value | 0.499 | 0.537 | 0.121 | 0.167 | 0.930 |
| Albumin |  |  |  |  |  |
| Correlation coefficient | 0.212 ^a^ | -0.279 ^a^ | 0.213 ^b^ | 0.148 ^a^ | -0.173 ^a^ |
| *P* value | 0.004 | < 0.001 | 0.004 | 0.045 | 0.036 |
| Calcium |  |  |  |  |  |
| Correlation coefficient | -0.112 ^a^ | -0.041 ^a^ | -0.024 ^b^ | -0.036 ^a^ | 0.044 ^a^ |
| *P* value | 0.122 | 0.620 | 0.743 | 0.616 | 0.587 |
| Phosphorus |  |  |  |  |  |
| Correlation coefficient | 0.264 ^a^ | -0.159 ^a^ | 0.230 ^b^ | 0.421 ^a^ | -0.200 ^a^ |
| *P* value | < 0.001 | 0.052 | < 0.001 | < 0.001 | 0.012 |
| Creatinine |  |  |  |  |  |
| Correlation coefficient | 0.357 ^a^ | -0.290 ^a^ | 0.447 ^b^ | 0.549 ^a^ | -0.255 ^a^ |
| *P* value | < 0.001 | < 0.001 | < 0.001 | < 0.001 | < 0.001 |
| Kt/V |  |  |  |  |  |
| Correlation coefficient | -0.115 ^a^ | 0.029 ^a^ | -0.107 ^b^ | -0.467 ^a^ | 0.092 ^a^ |
| *P* value | 0.136 | 0.742 | 0.168 | < 0.001 | 0.286 |

Notes: ^a^ Pearson’s correlation analysis; ^b^ Spearman’s correlation analysis

*Abbreviations: 6MWT* six-minute walk test, *STS10* 10-repetition sit-to-stand-to-sit test, *STS30* 30-second sit-to-stand-to-sit test, *HGS* handgrip strength, *TUG* timed up-and-go test, *BMI* body mass index
